# Cardiorespiratory fitness mediates cortisol and lactate responses to winter and summer marches

**DOI:** 10.5937/jomb0-44369

**Published:** 2024-01-25

**Authors:** Deniel Pešić, Mirjana M. Đukić, Ivan Stanojević, Vladimir Živkovć, Sergey Bolevich, Stefani Bolevich, Vladimir Jakovljević

**Affiliations:** 1 Military Medical Academy, Institute of Hygiene, Department of Exercise Physiology, Belgrade; 2 University of Belgrade, Faculty of Pharmacy, Department of Toxicology, Belgrade; 3 Military Medical Academy, Institute of Medical Research, Belgrade; 4 University of Kragujevac, Faculty of Medical Sciences, Department of Physiology, Kragujevac; 5 Center of Excellence for Redox Balance Research in Cardiovascular and Metabolic Disorders, Kragujevac + First Moscow State Medical University I. M. Sechenov, Department of Pharmacology, Moscow, Russia; 6 First Moscow State Medical University I. M. Sechenov, Department of Pharmacology, Moscow, Russia

**Keywords:** acclimatisation, cardiorespiratory fitness, cate cholamines, Cory cycle, cortisol, glycolysis, gluconeogenesis, lactate, marching, physical activity, thermoregulation, VO2MAX, aklimatizacija, fizička aktivnost, glikoliza, gluko neogeneza, kateholamini, kardiorespiratorna kondicija, Cori ciklus, kortizol, laktat, marširanje, metabolizam glukoze, VO2MAX

## Abstract

**Background:**

The influence of homeostatically regulated physiological processes, including cardiorespiratory fitness (VO2max), on the response to physical stressors such as acclimatisation and marching, remains understudied. We aimed to investigate the effects of summer and winter acclimatisation and marching on cortisol levels and blood lactate, to gain insight into the role of these physiological processes in the stress response.

**Methods:**

Two groups of young Europeans, classified as poor (PCF; n=9) and good physical condition (GCF; n=21), based on a VO2MAX threshold of 40 mL O2/ kg/min, underwent 2-h March (6-7 km/h) in winter (5˚C) and summer (32˚C). Commercial tests, UniCel DxI Access Cortisol assay and EKF Biosen Clinic/GP assay were used for cortisol and lactate blood measurements (morning samples and those taken immediately after marches), respectively.

## Abstract (sr)

**Uvod:**

Uticaj homeostatski regulisanih fizioloških procesa, uključujući kardiorespiratornu kondiciju (VO2max), na odgovor na fizičke stresore poput aklimatizacije i marširanja, ostaje nedovoljno proučen. Cilj istraživanja je bio i da se ispita uticaj aklimatizacije i marširanja tokom leta i zime na nivoe kortizola i laktoze u krvi, pružajući uvid u ulogu ovih fizioloških procesa u odgovoru na stres.

**Metode:**

Dve grupe mladih Evropljana, klasifikovane kao osobe sa slabom (PCF; n=9) i dobrom fizičkom kondicijom (GCF; n=21), na osnovu praga VO2MAX od 40 mL O2/kg/min, su bile izložene dvosatnom maršu (6-7 km/h) u dva navrata, zimi na 5°C i leti, na 32°C. U uzorcima krvi (jutarnji i neposredno nakon marša) su određivani nivoi kortizola i laktata komercijalnim testovima, UniCel DxI Access Cortisol i EKF Biosen Clinic/GP.

**Rezultati:**

Bazalni nivoi kortizola su bili značajno viši na 5°C nego na 32°C (u PCF grupi: P=0,0079); nakon marširanja na 5°C kortizol opada u obe grupe a povećan je na 32°C samo u PCF grupi. Bazalni nivoi laktoze bili su viši na 32°C samo u GCF grupi (poredeći sa PCF grupom, P=0,0014) i nakon marširanja (poredeći sa bazalnim vrednostima, P=0,0002).

**Zaključak:**

Za osobe sa lošijom fizičkom kondicijom, hladnoća je veći stres od vrućine, bazirano na višim nivoima bazalnog kortizola. Anaerobni metabolizam pre vladava kod osoba sa boljom fizičkom kondicijom na visokoj ambijentalnoj temperaturi, bazirano na višim baznim nivo ima i posle marširanja. Studija je značajna za indidvidualnu optimizaciju treninga/performansi jer ukazuje na povezanost fizioloških odgovora na stres i individualne fizičke kondicije.

## Introduction

Environmental temperature has a profound impact on the physiological response of humans during physical activity [1]. Acclimatisation refers to the organism's adaptation to extreme external temperatures and is mediated by the sympathetic nervous system (SNS) and the hypothalamic-pituitary-adrenal (HPA) axis, with cortisol secretion playing a pivotal role in the body’s stress response [1] [2]. Thermo regulation is primarily governed by the stimulation of adrenergic and cholinergic pathways and hormonal regulation predominantly mediated by cortisol and pancreatic hormones as well as energy metabolism, to ensure the necessary energy for maintaining internal temperature and optimal blood glucose levels [3] [4] [5].

Cortisol, a primary stress hormone, exerts significant influence over multiple physiological processes. It is integral to energy metabolism through gluconeogenesis (GNG) and plays a regulatory role in immune responses [6] [7] [8].

Lactate, or lactic acid, is a byproduct of anaerobic glycolysis and serves as a fuel source under anaerobic conditions. Intense and prolonged exercise increases lactate production due to the restricted oxygen (O_2_) supply at the cellular level until aerobic metabolism switches to anaerobic, commonly known as the lactate threshold [9].

Marching, a type of weight-bearing exercise, falls under acute and low to high-intensity exercise. Itis an energy-demanding activity that can be performed under aerobic or anaerobic conditions. Inresponse to physical exercise, glucose metabolic pathways such as GNG [synthesis of glucose from non-hexose compounds derived from carbohydrates (such as lactate, pyruvate, and glycolytic intermediates), lipids (free fatty acids), and proteins (amino acids)] and the Cori cycle (refers to the conversion of lactate produced in active skeletal muscles to glucose via hepatic GNG) are activated to meet the increased energy demands [9] [10] [11].

Individual’s physical condition, as indicated by cardiorespiratory fitness, particularly measured by VO2max (the maximum O_2_ consumption during exercise), may influence the stress response to physical exercise like marching. Higher VO2max is associated with better aerobic capacity and greater endurance performance, although there is limited evidence on the relationship between VO2max, acclimatisation, and energy metabolism, primarily glucose [12] [14] [13].

Given these considerations, the study aimed to evaluate the cortisol and lactate levels in young, healthy Caucasians (Europeans) descent with good (GCF) and poor (PCF) cardiorespiratory fitness and their relationship to thermoregulation and energy metabolism before and after a 2–3-hour march conducted in both summer (32°C) and winter (5°C) conditions.

## Materials and methods

### Administrative study procedures

All participants gave written informed consent after being informed of the purpose of the study, the test conditions, and the procedure. The study was conducted following the Declaration of Helsinki and approved by the Ethics Committee of the Military Medical Academy in Belgrade (No. 3/2019, dated May 16, 2019).

### Subjects

Thirty healthy male adults, aged 29.5 ± 6.0 years, with a body mass index (BMI) of 26.09 ± 2.89 kg/m^2^, white race, European, participated in the study. Participants were informed in detail about the study and gave written informed consent. They did not take any therapies or supplements, ate a regular diet with no special dietary restrictions, and exercised daily under aerobic and anaerobic conditions (long-distance running, football, basketball, workout, and strength exercises). According to a study by Lavenne et al. in 1966, individuals can be categorized as »high responders« or »low responders« to physical exertion in high ambient temperatures based on a threshold of 40 mL O_2_/kg/min [12]. Hence, we divide our participants into two groups: GCF group (good cardiorespiratory fitness) - n=9 (VO2max <40 mL O_2_/kg/min) and PCF (poor cardiorespiratory fitness) - n=21, (VO2max >40 mL O_2_/kg/min).

### Study design

All participants marched an ~ 15-km route at an average speed of 6–7 km/h on relatively flat terrain (combination of asphalt and dirt road, with no significant inclines; at latitude 44° N and elevation of 80–100 m above sea level) for approximately 2 hours, beginning in the morning hours at ~ 10 am. Participants marched the same route twice, in winter (November, 5°C) and in summer (August, 32˚C).

They wore appropriate athletic clothing, carried a load equivalent to 5–10% of their body weight to simulate marching soldiers and were allowed to drink water during the march. In addition, the health status of the participants was continuously monitored during the march on both occasions (winter and summer marches).

### Blood sampling

Blood lactate and cortisol levels were measured in the morning hours (10 am) before the marches and served as baseline values.

For cortisol measurements, blood samples were taken from vena Cubitalis (in the nearby laboratory, immediately before and immediately after marches (without any intermediate pause, while the participants’ pulse had not yet settled down).

Capillary blood sampling (0.1 mL) for lactate analysis was taken »in the field« before and immediately after marches.

### Cortisol measurement

Cortisol was analysed by a standard nonisotopic heterogeneous competitive immunoassay (UniCel DxI Access Cortisol assay), the carbonyl metalloimmunoassay (CMIA), using 50 mL of serum, performed at UniCel DxI Access Immunoassay Systems (a commercial analyser; manufacturer: Beckman Coulter, the United States). Intra-assay CVs ≤2.5%, and interassay CVs ≤2.8% [15].

### Lactate measurement

The commercial test»EKF Biosen Clinic/ GP« was performed for lactate analysis on BIOSEN C line by EKF Diagnostics (a commercial analyser; manufacturer: EKF Diagnostics, Germany). The measuring range of the method for lactate is 0.5–40 mmol/L; imprecision: CV ≤1.5% (12 mmol/L).

The principle of the test is based on the electrochemical measurement (with chip sensor electrodes) of hydrogen peroxide, the product of the oxidation of β-D-glucose/L-lactate to d-glucono-lactone/pyruvate by glucose oxidase/lactic acid oxidase in the presence of O_2_ [16] [17].

### Statistical data analysis

Data analysis was performed using GraphPad Prism 6 software. Kolmogorov-Smirnoff normality tests and D’Agostino and Pearson omnibus normality tests were used to test the normality of the data distribution. Then, the appropriate parametric (Student’s t-test for unpaired data and paired t-tests for paired data) or nonparametric (Mann Whitney test for unpaired data and Wilcoxon test for paired data) tests and Pearson correlation tests or Spearman correlation tests were used for statistical data analysis. Significance was assumed at a confidence level of P <0.05.

## Results

The results of serum cortisol and lactate levels for all participants (both groups, GCF and PCF) are presented tabularly (basic descriptive statistics) and graphically (statistical significance was shown).Table 1

**Table 1:** Blood cortisol and lactate levels of all participants. Basal values (B) of serum cortisol (nmol/L) and lactate (mmol/L) (blood samples taken immediately before the march, at 10 am); lactate values in the march (M) (blood samples taken immediately after the march, while the participants’ pulse had not yet settled). Participants (young male adults, white race and European) were divided into the group with good cardiorespiratory fitness (GCF): n=9 (VO2max < 40 mL O_2_/kg/min) and the group with poor cardiorespiratory fitness ( PCF ): n=21, (VO2max > 40 mL/kg/min); participants wore the same sportswear, equipment, and were loaded with gear weighing 5–10% of their body mass (to simulate marching soldiers), and were allowed to drink water during the march (15 km, average speed of 6–7 km/h, latitude ~ 44˚ N, altitude of 80-100 m, on flat terrain); the march was performed twice, in winter (November, at 5˚C) and in summer (August, at 32˚C).

		Cortisol (nmol/L)				Lactate (mmol/L)			
		32 °C		5 °C		32 °C		5 °C	
		Basal	Marching	Basal	Marching	Basal	Marching	Basal	Marching
GCF (n=21)	Average	307.3	318.3	381.9	248.1	3.367	5.195	2.331	2.456
	St. dev.	72.72	130.8	149.7	131	1.141	1.829	0.7844	0.6283
	St. error	16.26	29.24	34.35	30.05	0.2551	0.409	0.1961	0.1571
PCF (n=9)	Average	302.7	347.2	374.3	221.5	2.765	3.706	2.788	2.635
	St. dev.	60.2	72.89	68.01	92.1	0.8532	1.216	2.093	1.237
	St. error	20.07	25.77	22.67	32.56	0.3017	0.4053	0.74	0.4372

**Figure 1:**
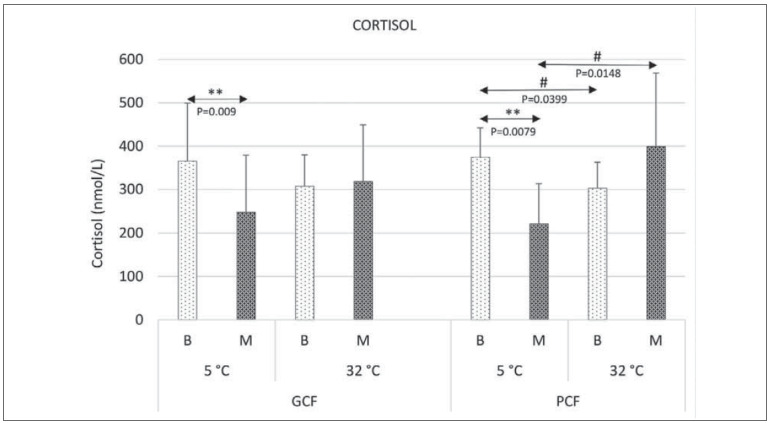
Basal blood cortisol levels immediately after marching at 5°C and 32°C in GCF and PCF participants, respectively. Serum cortisol (nmol/L): basal blood cortisol values refer to morning values (B)-blood samples taken during marching hours (around 10 am) immediately before marching; immediately after marching, lactate values (M) (blood samples taken immediately after marching, while participants’ pulse had not yet settled). Participants (young male adults, Caucasian and European) were divided into a group with good cardiorespiratory fitness (GCF): n=9 (VO2max <40mL O_2_/kg/min) and a group with poor cardiorespiratory fitness (PCF ): n=21, (VO2max >40 mL/kg/min); participants wore the same sports clothing, equipment, and were loaded with equipment weighing 5–10% of their body mass (to simulate marching soldiers) and were allowed to drink water during the march (15 km, average speed of 6–7 km/h, latitude ~ 44˚ N, altitude of 80–100 m, on flat terrain); the march was performed twice, in winter (November, at 5˚C) and in summer (August, at 32˚C); the statistical confidence level: significance was set at P <.05. Symbols for significance: *refers to the values obtained for one group and one temperature (5˚C and 32˚C); # refers to the values obtained for one group and different temperatures. Marking of statistical significance: # P <.05; ** P <.01.

A significant difference between basal cortisol levels at 5°C and 32°C was found only in the PCFgroup (at 5°C, 374.3±68.01 nmol/L and at 32°C, 302.7±60.2 nmol/L; P=0.0399); no significance was found for GCF, at 5°C, 365.0±134.3 nmol/L and at 32°C, 307.3±72.72 nmol/L (Figure 1).

After marching at 5C, cortisol decreased significantly in both groups: in the GCF group (to 248.1±131 nmol/L, P=0.009); and in the PCF group (to 221.5±92.1 nmol/L, P=0.0079) (Figure 1).

After marching at 32°C, cortisol increased slightly (not significantly) in both groups, although theresponse in the PCF group was significantly higher than that at 5°C (P=0.0148) (Figure 1).

**Figure 2:**
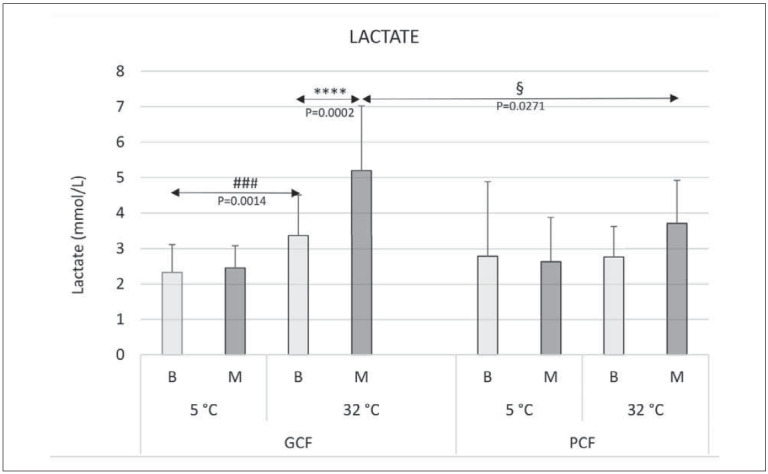
Basal blood lactate values immediately after the march at 5°C and 32°C in GCF and PCF participants, respectively. Serum lactate (mmol/L). For further details, see the legend in Figure 1. Symbols for significance: *refers to values obtained for one group and one temperature (5˚C and 32˚C); # refers to values obtained for one group and different temperatures; § refers to comparingvalues between groups GCF and PCF and for different temperatures. Labeling of statistical significances: § P <.05; ### P <.005; **** P <.0005.

Basal blood lactate levels were significantly higher at 32°C (3.4±1.14 mmol/L) than at 5°C (2.34±0.78 mmol/L) in the GCF group (P=0.0014). Within the PCF group and compared with the GCF group, no significant difference was observed between basal blood lactates at 5°C and 32°C (Figure 2).

After marching at 5°C, no significant changes in blood lactate levels were observed in either group (Figure 2).

After marching at 32°C, blood lactate increased significantly by 35% (P=0.0002) from 3.4±1.14mmol/L to 5.2±1.83 mmol/L in the GCF group only; moreover, lactate levels after marching at 32°C were significantly higher in the GCF group than in PCF (P=0.0271); moreover, an insignificant increase in blood lactate (from 2.8±0.85 mmol/L to 3.7±1.22 mmol/L (Figure 2) occurred in the PCF group after marching at 32°C.

## Discussion

The study contributed several significant findings: (i) basal blood cortisol levels were higher duringcold exposure than exposure to higher ambient temperatures in all participants (with significance achieved in PCF group, P=0.0079), suggesting that cold weather elicits a greater physiological stress response particularly for individuals with worse physical fitness; (ii) a novel finding was the association between heat exposure and increased blood lactate levels, only in GCF participants (P=0.0014), implying that the lactate response to heat stress may be influenced by individual physical fitness; (iii) following march at 5°C, all participants exhibited significantly lower cortisol levels compared to their basal levels (GCF: P=0.009, PCF: P=0.0079) indicating that physical activity in cold temperatures may have a stress-reducing effect on cortisol levels, irrespectively of physical condition; (iv) PCF participants experienced higher cortisol levels after marching at 32°C, though insignificantly; (v) GCF participants exhibited a significant increase in blood lactate levels following the march at 32°C (P=0.0002), only, indicating that higher temperatures may lead to greater lactate production during exercise in individuals with better physical fitness.

These results highlight the differential cortisol and lactate responses to physical stressors (acclimatisation and marching) concerning individual physical conditions.

### General discussion

The general discussion of the study findings highlights the significant contributions and implications of the research. The study revealed several crucial findings related to cortisol and lactate responses to different physical stressors, such as exposure to cold and heat and physical exercise. The close interaction between the adrenergic and cholinergic pathways is also highlighted in the study. These pathways play a vital role in modulating cortisol and lactate levels through their effects on hemodynamics and energy metabolism. The intricate interplay between these pathways underscores their influence on the body’s stress response and energy regulation.

The regulation of basal cortisol and lactate levels during heat, cold, and exercise is influenced by individual VO2max. It involves changes in vascular tone, energy metabolism, and glucose metabolism regulated by cortisol, insulin, glucagon, and catecholamines [11] [14] [18] [19] [20] [21]. The type and duration of outdoor physical exercise dictate maintain of internal body temperature (thermoregulation) and a degree of O_2_ consumption; therefore, the mode of glucose metabolism (aerobic or anaerobic), which are the two critical homeostatic mechanisms discussed herein [22].

Cortisol is a stress hormone reflecting the body’s response to various stressors, including physical stressors like extreme ambient temperatures and exercise [1] [23].

Cortisol and lactate are two different biomolecules that emerged from different physiological processed and are related to stress responses such as acclimation and energy metabolism [24]. Cortisol, an essential human stress hormone, and lactate (a byproduct of anaerobic glycolysis) reflect neurohumoral mechanisms of thermoregulation and energy metabolism in individuals exposed to outdoor exercising, respectively, which are, at the same time, the critical underlying homeostatic processes engaged in the overall response to physical stimuli to which our participants were exposed [6] [8] [22] [24] [25] [26].

### Acclimatisation/thermoregulation

Acclimatisation to cold stress involves physiological and acclimation responses within the first few days up to three weeks. This includes habituation (subdued shivering and cutaneous vasoconstriction), metabolic adaptations (increased thermogenesis, except in extreme cold exposure leading to hypothermia), and insulating adaptations (increased vasoconstriction and redistribution of body heat to the periphery, potentially causing hypothermia) [2] [27].

On the other hand, heat stress adaptations typically occur within 7–10 days, with well-trained athletes adapting faster [28] [29]. Humans have likely developed an efficient thermoregulatory system against cold, supported by thermoreceptor localisation, cold thermal zones, and faster afferent conduction [18] [30].

### Transmission of cold and hot sensation

The human thermoregulatory system perceives cold temperatures as more significant stress than heat. Thermoreceptors responsible for cold and heat sensation differ in morphology and distribution, primarily found in the skin rather than skeletal muscles, explaining the absence of differences in cortisol response between individuals with high and low cold tolerance [18] [26] [31] [32] [33]. Thermoreceptor density varies based on factors like age, sex, and health status [6] [34]. Cold sensations are transmitted rapidly to the hypothalamus through myelinated A-fibers, while slower C fibres relay heat sensations. Both A and C fibres supply cold thermoreceptors, while warm thermoreceptors are solely supplied by C fibres [35] [36] [37].

### Stimulation of adrenergic and cholinergic pathways

The involvement of cortisol and catecholamines, mediated through the activation of the SNS and the HPA axis, plays a critical role in the regulation of the body’s internal temperature. The adrenergic and cholinergic pathways intricately influence cortisol and lactate levels in the human body through their effects on hemodynamics and energy metabolism. During cold exposure, the HPA axis stimulates the adrenal cortex to secrete cortisol, dehydroepiandrosterone, and aldosterone, impacting metabolism. The adrenal medulla releases catecholamines like epinephrine (E), norepinephrine (NE), and dopamine (DA) [8] [11] [19] [20] [21] [38] [39] [40] [41] [42].

The sympathetic nervous system (SNS) releases NE, DA, and acetylcholine (ACh) at effector organlevels, affecting smooth muscles, the heart, and glands [43]. Noradrenaline predominantly stimulates alpha-adrenergic receptors (α1-ARs, α2-ARs), while E predominantly activates beta-adrenergic receptors (β1-ARs, β2-ARs, β3-ARs) [43].

### Cold exposure

Catecholamines induce hemodynamic effects by acting on adrenergic receptors in the smooth muscles of blood vessels. During cold exposure, hemodynamic adrenergic effects (signalling from the thoracolumbar region of the spinal cord) refer to peripheral (via NE-stimulated alpha1-ARs in larger vessels and normally inactive alpha2-ARs in cutaneous arteries), systemic vasoconstriction (through α2C-ARs stimulation) increasing systemic vascular resistance and enhancing heart muscle contraction, rate, and relaxation (via E stimulation of β1-ARs, mainly present in the heart) [44] [45] [46].

Catecholamines influence energy metabolism, especially glucose metabolism, making the investigation of lactate levels relevant in evaluating energy metabolism during acclimatisation and physical exercise [47]. Adrenergic signalling pathways contribute to vasoconstriction (help conserve heat by reducing blood flow to the extremities) and shivering (heat production in skeletal muscles), respectively [44] [45] [46].

Catecholamines support energetic metabolism by promoting glycogenolysis and glucose-6-phosphatase (G6Pase)-mediated glucose production through NE stimulation of hepatic α1-ARs and E-stimulated hepatic β2-ARs, respectively, which contribute to the increase in internal body temperature during cold exposure. Glycogenolysis in skeletal muscles is also facilitated by the stimulation of β2-ARs by adrenaline [48]. Also, E-stimulated β3-ARs in brown adipose tissue induce thermogenesis and lipolysis [49].

Cholinergic pathways (from the sacral region of the spinal cord) play a crucial role in the shivering response to cold exposure (ACh-mediated skeletal muscle tremors via stimulation of nicotinic receptors). Shivering generates heat, helping to maintain body temperature, regardless of an individual’s physical condition [43].

### Heat exposure

Elevated ambient temperatures activate β2-ARs through E, causing relaxation of smooth muscles,including cardiac and skeletal muscles, as well as peripheral vasodilation [48]. Peripheral vasodilation enhances body heat dissipation through convection and evaporation, facilitated by increased blood flow, allowing for faster heat transfer from the core to the skin. NE-stimulated α2-ARs in the synaptic cleft decrease sympathetic efferent signals, resulting in positive inotropic effects, muscle relaxation (including cardiac and skeletal muscles), reduced venous volume returning to the heart, increased stroke volume, and heart rate to maintain systemic blood pressure (compensatory feedback loop).

In terms of energy metabolism, NE inhibits lipolysis in adipose tissue via α2-ARs during heat exposure, and excess heat is released through sweating via cholinergic stimulation (from the thoracic splanchnic region) of sweat glands muscarinic receptors, releasing excess internal heat [50] [51] [52]. Also, insulin secretion increases while glucagon secretion decreases, confirming reduced energy demand during exposure to hot weather [53] [54].

This increased blood flow benefits active skeletal muscles, promoting endurance during activities like marching [53] [55] [56] [57] [58].

### Glucose metabolism and lactate

### Glucose metabolism

Glucose metabolism and lactate play vital roles in energy regulation during physical exercise, with different processes and pathways contributing to their utilisation and homeostasis. Glucose metabolism is crucial for maintaining optimal blood glucose levels during acclimation or physical exertion [21]. It involves hormonal control and adrenergic stimulation from the sympathetic nervous system [59] [60]. Glycolysis, gluconeogenesis (GNG), glycogenesis, and glycogenolysis are opposing processes in glucose metabolism [61] [62]. regulated based on the body’s needs and health conditions.

Glycolysis breaks down glucose into pyruvate, generating ATP and NADH+H^+^ [21] [63]. Pyruvate can undergo aerobic glycolysis in the presence of oxygen, producing acetyl CoA for the Krebs cycle and more ATP.

### Lactate

In the absence of oxygen, anaerobic glycolysis takes place (converts pyruvate to L-lactate using lactate dehydrogenase and NADH+H^+^ as cofactors), producing 2 ATP per glucose molecule. This pathway is significantly faster than oxidative phosphorylation and increases lactate production during intense exercise [6] [64]. The formation of lactate under fully aerobic conditions of rest and exercise represents an important mechanism by which different tissues share a carbon source (lactate) for oxidation and other processes such as GNG. This mechanism has been termed the lactate shuttle. Lactate production under fully aerobic conditions: the lactate shuttle during rest and exercise [47].

During intense exercise, anaerobic glycolysis is relied upon to meet the energy demand, leading tolactate production [6] [64]. Lactate serves as a carbon source for oxidation and other processes like gluconeogenesis [47]. It can be utilised as a respiratory fuel or for glycogen re-synthesis in skeletal muscle and the heart [24] [65] [66] [67].

Lactate homeostasis is maintained through the interconversion of glucose and lactate, contributing to energy metabolism [11] [68] [69] [70] [71]. Cells with mitochondria produce lactate under anaerobic conditions, while non-mitochondrial cells produce lactate under normal physiological conditions [6] [64]. Lactate also regulates energy metabolism by affecting blood pH sensed by chemoreceptors [72].

### Marching, VO_2_ max and energy metabolism

The Cori cycle is essential to energy metabolism during heavy exercise [73] [74]. In active skeletal muscle, glycogenolysis occurs via β2-adrenergic receptor (β2-AR) stimulation, while in the liver, it proceeds further to release glucose into the bloodstream [73] [74]. The activation of α1-adrenergic receptors promotes glycogenolysis via G1Pase stimulation in muscle. Hepatic glycogenolysis ensures the release of glucose to meet the body’s energy requirements and maintain blood glucose levels. Glycogenolysis stops at G6P in muscles, which can be used for immediate glycogenesis [75] [76] [77] [78]. Glucagon, which increases during exercise, stimulates hepatic glycogenolysis and GNG while inhibiting glycolysis, glycogenesis, and lipogenesis [49] [79]. In contrast, insulin promotes glucose uptake, glycolysis, and glycogenesis and inhibits GNG [76] [80] [81] [82]. During moderate exercise, aerobic glycolysis produces additional acetyl-CoA for energy [83].

Physical activity requires additional energy, and glucose metabolism, including glycolysis, GNG, glycogenolysis, and the Cori cycle, play crucial roles in providing energy [11] [12].

Padgett DA, Glaser R. [84] found that higher VO2max is associated with greater muscle mass,improved blood circulation, and lower inflammatory markers, indicating better adaptation to exercise. It is also suggested that higher VO2max enhances glucose transport and utilisation in working muscles, prolongs aerobic exercise duration due to good blood flow, and elevates the lactate threshold compared to individuals with lower fitness levels [85]. However, exercise-induced oxidative stress can increase inflammation and cortisol production [86]. Gregg SG. et al. [85] reported a positive correlation between higher energy demands and glucose metabolism rate in trained athletes, supporting our findings of elevated lactate levels in GCF participants, particularly during the summer season (Figure 1).

Blaxter K. [39] found that untrained individuals typically have lower muscle glycogen stores compared to endurance athletes. This can lead to higher perceived exertion, increased cortisol release, and a greater reliance on anaerobic metabolism, resulting in elevated stress and cortisol levels [39]. On the other hand, Marcinik EJ et al. [87] showed that strength training improves physical endurance performance independent of changes in VO2max and is associated with improvements in lactate threshold (LT) and leg strength. Similarly, Aagaard P et al. [88] found that combining strength and endurance training in young elite competitive cyclists led to enhanced endurance capacity, increased type IIA muscle fibres, maximum voluntary contraction, and rate of force development. Additionally, Wolfarth B. et al. [29] highlighted the influence of genetic factors on VO2max, particularly the importance of polymorphisms near α2A-ARs genes for better endurance status in warm weather among elite athletes. However, the study of Gorski and De Bock [89] highlighted the crucial role of blood vessel networks in supplying O_2_ and nutrients to skeletal muscle, contributed by skeletal muscle angiogenesis and its influence on exercise performance.

### Discussion of the obtained results: Basal values of cortisol and lactate at 5°C and 32°C.

### Basal cortisol at 5°C

Higher basal cortisol levels were consistently observed in all participants during cold exposure compared to exposure to higher outside temperatures, with significance achieved in the PCG group (P=0.0399) aligning with the physiological principles of thermoregulation. Our results are also consistent with the study conducted by Pääkkönen T and Leppäluoto J [8], which reported a more profound hormonal response (elevated basal cortisol levels) during cold exposure, providing further support for the perception of cold as a greater threat than hot weather [1]. In the context of cold exposure, cortisol production and secretion are enhanced through dual stimulation involving the HPA axis as well as the cholinergic thoracic splanchnic region of the spinal cord [90] [91].

Cortisol levels during cold exposure are regulated through a negative feedback mechanism connected to energy metabolism [11] [21] [48]. Glycogen is primarily stored in skeletal muscles, with a smaller extent in the liver, and typically lasts around 24 hours. This duration corresponds to the natural circadian rhythm, which involves daily and monthly fluctuations in cortisol secretion, optimising brain function regulated by the autonomic nervous system and cardiovascular system. Accordingly, morning basal cortisol levels did not significantly differ between the GCF and PCF groups in our study participants (Figure 1). It’s noteworthy that Central Europeans often experience a peak in cortisol levels during marching [20] [40] [41].

### Basal cortisol at 32°C

The explanation for the lower basal cortisol levels in the summertime is that heat exposure triggers a less intense stress response than cold exposure, based on the adaptation mechanisms to heat stress (Figure 1) [34] [72]. During heat stress, the body does not require extra energy, as indicated by increased insulin secretion, decreased glucagon secretion (aiming to remove excess glucose from the blood), and reduced stimulation of cortisol secretion. This is mediated by negative feedback regulation between blood glucose levels and cortisol secretion [3]. Namely, the HPA axis is not stimulated; thus, cortisol (and E) secretion primarily relies on cholinergic stimulation from the thoracic splanchnic region of the spinal cord [91]. However, via feedback mechanisms of glucose blood levels, Increased insulin secretion and decreased glucagon secretion contribute to lower blood glucose levels, indicating that additional energy is not needed during heat stress [24] [60].

### Basal lactates at 5°C

Basal lactate levels decrease during cold exposure due to decreased metabolic rate and increased energy demands for maintaining body temperature, as well as systemic and peripheral vasoconstriction [31]. In PCF participants, basal lactate levels were not significantly different between 5°C and 32°C, but slightly higher lactate levels were found at 5°C, consistent with the findings of Goldstein et al. [46].

### Basal lactates at 32°C

At 32°C, GCF participants showed significantly higher basal lactate levels due to vasodilatory effects, increased metabolic rate, suppressed cortisol-stimulated GNG, and improved blood flow [92].

### Post-march values of cortisol and lactate at 5°C and 32°C

### Lactates after marching at 5°C

During marching in cold weather, lactate levels remained stable in all participants. This stability can be attributed to the restriction of lactate release, which is caused by a reduction in the Cori cycle and GNG. This, in turn, leads to a decrease in anaerobic glycolysis as an energy source. As a result of these processes, the lactate threshold is slightly surpassed.

Furthermore, the increased utilisation of pyruvate for aerobic metabolism may contribute to controlled lactate production, preventing significant accumulation during exercise in cold weather [6] [11] [12] [64] [76]. This aligns with the findings of Brooks GA. [47] showed that lactate production and oxidation increase, but to a lesser extent compared to O_2_ consumption, during moderate-intensity exercise, indicating sufficient oxygenation of skeletal muscles. Even in the presence of adequate O_2_, certain muscles still produce lactate through glycolysis. Lactate formation allows different tissues to share a carbon source for oxidation and other metabolic processes, such as GNG, under aerobic conditions. This process is known as the lactate shuttle [47].

It is worth noting that lactate accumulation is more dependent on exercise intensity and duration rather than cold stress alone, according to De Carvalho FG’s study in 2003 [93]. The consistent lactate values observed in our study after marching at 5°C align with the findings of MacRae et al. [94] study demonstrated a decrease in lactate blood levels at lower work rates and an increase in lactate metabolic clearance at higher work rates after training, indicating enhanced lactate utilisation during exercise.

### Lactates after marching at 32°C

Post-march lactate levels were significantly higher in all subjects at 32°C, which is consistent with the findings of Karlsen et al. [95] that heat acclimation in well-trained cyclists alters muscle metabolism without performance improvements compared to untrained individuals. However, in the GCF group, both basal lactate values (basal lactate levels were significantly higher at 32°C than at 5°C, P=0.0014) and after the march (lactate levels were significantly higher after marching form basal values at 32°C, P=0.0002) may be attributed to higher exercise capacity, metabolic demand, oxidative capacity, more developed and vascularised muscles, more intense marching activities, higher blood flow, improved heat dissipation, and subsequent lactate release into the bloodstream in GCF participants [55] [57].

The elevated lactate levels observed in GCF participants can be attributed to the combination ofenhanced energy metabolism in well-trained individuals and the activation of α2A-ARs and β2-ARs during heat stress [29]. Genetics play a crucial role in determining maximal aerobic power and trainability, with specific polymorphisms near the α2A-ARs genes being associated with improved endurance status in warm weather among elite athletes, according to Wolfarth B. et al. [29].

Several factors contribute to the higher lactate levels observed in GCF participants. Firstly, both basal lactate values and lactate levels after the march were higher in GCF participants. This can be attributed to their well-developed and vascularised muscles and engagement in more intensive marching, leading to vasodilation and increased lactate production. The elevated lactate levels in GCF participants can be attributed to their higher energy metabolism and the activation of α2A-ARs and β2-ARs during heat stress, promoting vasodilation, bronchodilation, muscle relaxation, and glycogenolysis [96]. This, in turn, increases blood flow to skeletal muscles and facilitates lactate release, as supported by Emhoff Chi-An W. et al. [97] (Figure 2).

These findings are consistent with our hypothesis that hot weather exercise increases heat stress and favours anaerobic metabolism, particularly in GCF participants, highlighting the novel aspect of this study.

One possible explanation for the elevated lactate levels in GCF participants following marching inhigh temperatures could be attributed to increased skeletal muscle lactate production due to intensified anaerobic metabolism and reduced GNG, as cortisol stimulation is not observed in hot environmental conditions [97] [98]. A study by Emhoff Chi-An W. et al. [97] in 2013 found that lactate oxidation rates significantly increased during exercise compared to rest. Trained individuals exhibited a higher proportion of direct lactate oxidation, suggesting training adaptations and a potential glycogen-sparing effect in the muscles during exercise.

### Cortisol after marching at 5°C

The decrease in cortisol levels is statistically significant in both GCF and PCF participants (P=0.009 in GCF and P=0.0079 in PCF). By benefiting from their improved aerobic capacity and enhanced heat dissipation through sweating, GCF participants experience lower cortisol levels and reduced stress during exercise in low ambient temperatures. Individuals with low VO2max may exhibit higher stress and cortisol levels under similar conditions [99] [100].

These observations are supported by the study conducted by Vuorimaa T. [100] in 2007, which demonstrated that prolonged low-intensity exercise in well-trained athletes does not result in elevated levels of oxidised LDL-cholesterol or oxidative stress. Furthermore, the study by Eijsvogels TMH [101] in 2011 found a positive correlation between cortisol levels and oxidative stress, further supporting our findings.

### Cortisol after marching at 32°C

Our study confirmed the expected pattern of stress levels decreasing during a winter march and increasing during a summer march [102]. Physical exercise in hot weather increases the body’s heat load due to increased metabolic heat production, leading to slightly elevated cortisol levels after marching in both GCF and PCF groups [99]. This supports the hypothesis that heavy exercise in hot weather causes additional stress, particularly in individuals with poor physical condition, as shown by comparing results after exercise at 5°C and 32°C (Figure 1) [99] [100]. GCF participants exhibited lower post-exercise cortisol levels in hot weather, attributed to their more efficient cardiovascular system, better heat dissipation, sweating, and maintenance of body temperature within a safe range, resulting in lower stress response and cortisol levels [85]. Additionally, the release of endorphins and other hormones during exercise may contribute to a better hormonal response and positive mood [103].

The higher cortisol levels among PCF participants after marching in summer (Figure 1) can beattributed to their emotional response to the activity, leading to heightened anxiety and perceived stress. This finding is in accordance with previous studies by Pelletier KR. [23] and Chow YW, et al. [104], suggesting that individuals approaching challenging situations with suspicion or uncertainty experience higher stress. This is particularly relevant for individuals with poor physical conditions, who tend to anticipate and approach physical stressors with heightened anxiety and stress levels, resulting in increased cortisol secretion.

## Conclusion

This study provides several significant findings that shed light on the differential cortisol and lactate responses to external physical stressors, including seasonal temperatures alone and when combined with physical exercise (a two-hour march) within the same environmental conditions concerning individual physical fitness.

The key findings about cortisol blood levels are as follows: (i) Basal blood cortisol levels were higher during cold exposure compared to exposure to higher ambient temperatures in all participants, indicating that cold weather elicits a more pronounced physiological stress response, particularly among individuals with poorer physical fitness. (ii) Following a march at 5°C, all participants displayed significantly lower cortisol levels relative to their baseline levels, suggesting that physical activity in cold temperatures may mitigate cortisol levels, irrespective of physical condition. (iii) Higher cortisol levels were observed after marching at 32°C in participants with poorer physical fitness, indicating that individuals with lower physical fitness perceive physical exercise in hot weather as more stressful compared to those with better physical fitness; (iv) a novel finding indicates that exposure to heat resulted in increased blood lactate levels in the GCF group, both at rest and following the march in hot weather, suggesting the occurrence of more intense and enhanced energy metabolism during heat exposure in individuals with better physical fitness.

Our findings highlight the influence of individual physical conditions on cortisol and lactate responses to stressors, emphasising the importance of acclimatisation, thermoregulation, and glucose metabolism. The study is significant for individual training/performance optimisation, highlighting the association between physiological stress responses and physical fitness. Further research is needed to understand underlying mechanisms and develop interventions for stress management and energy metabolism in different environments.

## Dodatak

### List of Abbreviations

Acetyl-CoA, acetyl-coenzyme A;<br>Ach, acetylcholine;<br>ADP, adenosine diphosphate;<br>ATP, adenosine triphosphate;<br>D, dopamine;<br>E, epinephrine (adrenaline);<br>F6P, fructose-6-phosphate;<br>G6P, glucose-6-phosphate;<br>GCF, good cardiorespiratory fitness;<br>GNG, gluconeogenesis;<br>HPA, hypothalamic-pituitary-adrenal axis;<br>NAD^+^, an oxidised form of nicotinamide adenine dinucleotide;<br>NADH, a reduced form of nicotinamide adenine dinucleotide (H for hydrogen);<br>NE, norepinephrine (noradrenaline);<br>PCF, poor cardiorespiratory fitness;<br>PDC, pyruvate decarboxylase;<br>PDH, pyruvate dehydrogenase complex;<br>SNS, sympathetic nervous system.

### Acknowledgements

The Ministry of Science funded this research, Technological Development and Innovation, Republic of Serbia, through a grant agreement with the University of Belgrade – Faculty of Pharmacy No: 451-03-47/2023-01/ 200161; and granted by the Ministry of Defense»Investigation of DP-3 distracting half-meal« (2018–2019). The authors thank colleagues Marijana Andjic and Nevena Draginic for the laboratory assistance.

### Conflict of interest statement

All the authors declare that they have no conflict of interest in this work.
